# A machine learning-based linguistic battery for diagnosing mild cognitive impairment due to Alzheimer’s disease

**DOI:** 10.1371/journal.pone.0229460

**Published:** 2020-03-05

**Authors:** Sylvester Olubolu Orimaye, Karl Goodkin, Ossama Abid Riaz, Jean-Maurice Miranda Salcedo, Thabit Al-Khateeb, Adeola Olubukola Awujoola, Patrick Olumuyiwa Sodeke

**Affiliations:** 1 Department of Health Services Management and Policy, College of Public Health, East Tennessee State University, Johnson City, TN, United States of America; 2 Psychiatry Research Division, Department of Psychiatry and Behavioral Sciences, Quillen College of Medicine, East Tennessee State University, Johnson City, TN, United States of America; Nathan S Kline Institute, UNITED STATES

## Abstract

There is a limited evaluation of an independent linguistic battery for early diagnosis of Mild Cognitive Impairment due to Alzheimer’s disease (MCI-AD). We hypothesized that an independent linguistic battery comprising of only the language components or subtests of popular test batteries could give a better clinical diagnosis for MCI-AD compared to using an exhaustive battery of tests. As such, we combined multiple clinical datasets and performed Exploratory Factor Analysis (EFA) to extract the underlying linguistic constructs from a combination of the Consortium to Establish a Registry for Alzheimer’s disease (CERAD), Wechsler Memory Scale (WMS) Logical Memory (LM) I and II, and the Boston Naming Test. Furthermore, we trained a machine-learning algorithm that validates the clinical relevance of the independent linguistic battery for differentiating between patients with MCI-AD and cognitive healthy control individuals. Our EFA identified ten linguistic variables with distinct underlying linguistic constructs that show Cronbach’s alpha of 0.74 on the MCI-AD group and 0.87 on the healthy control group. Our machine learning evaluation showed a robust AUC of 0.97 when controlled for age, sex, race, and education, and a clinically reliable AUC of 0.88 without controlling for age, sex, race, and education. Overall, the linguistic battery showed a better diagnostic result compared to the Mini-Mental State Examination (MMSE), Clinical Dementia Rating Scale (CDR), and a combination of MMSE and CDR.

## Introduction

Mild Cognitive Impairment due to Alzheimer’s disease (MCI-AD) is a precursor to Alzheimer’s disease (AD) [1–3]. It is characterized by a cognitive decline that is usually associated with aging or AD [4]. Some of the profound characteristics of MCI-AD are the gradual degrading of cognitive speech functions, which is often affected long before the diagnosis of MCI-AD [5]. Research has shown that neurodegenerative disease such MCI-AD deteriorates nerve cells that control cognitive speech and language processes, and therefore affect the ability of an individual to make effective verbal utterances [6, 7].

The need for early detection of MCI-AD using linguistic biomarkers has been growing in recent years [6–12]. However, it is still prevalent to diagnose MCI-AD using a combination of neuropsychological batteries and a doctor’s longitudinal observation of the individual [13]. A more popular option is to use the Mini-Mental State Examination (MMSE) instrument which looks for gross cognitive deficits that may not be sensitive enough to capture low-level cognitive deficits that often characterize MCI-AD [14, 15]. It is also common to combine the MMSE with another test battery [13]. The Montreal Cognitive Assessment (MoCA)[16], Clinical Dementia Rating Scale (CDR) [17], Boston Naming Test (BNT) [18], and the Consortium to Establish a Registry for Alzheimer’s disease (CERAD) battery [19], are often combined to diagnose MCI-AD. Administering these neuropsychological batteries can be lengthy and complicated [16]. At the same time, each test battery would typically assess multiple cognitive deficits. The clinician would then need to make a challenging determination of an ideal diagnosis by considering all the possible cognitive deficits linked to different variants of a neuropsychological disorder [20].

Some independent linguistic subtests are available as part of the existing test battery such as the CERAD battery [21]. Many of the CERAD linguistic subtests have been assessed or validated independently or as part of a combined battery [22]. What is often less studied, however, is the combination of multiple linguistic subtests to create an independent linguistic battery for early diagnosis of MCI-AD using language alone. An independent linguistic battery could reduce the test burden on patients over a large population while at the same time increases the effectiveness of the diagnosis even with less test time.

For example, the efficacy of the Wechsler Logical Memory (WLM) I and II, which are two of the five different subtests of the Wechsler Memory Scale (WMS) [23], have been reported by some researchers to independently screen MCI-AD patients through a narrative task [6, 15, 24]. The WLM involves two narrative tasks, where the participants listen to a tale and then retell the tale both immediately after listening to the story and after a delayed period of about 30 minutes. The immediate retell is known as the WLM I, and the delayed retell is known as WLM II. A famous tale that is often used for the clinical diagnosis of MCI-AD is the Anna Thompson story [15]. Each narrative of the participant is scored based on the number of story elements in the narrative. The WLM I and II give a single summary score for retelling the narrative, immediate memory, and delayed memory [11, 15, 21, 24].

Similarly, the CERAD Word List Memory subtest, which is a part of the CERAD neuropsychological assessment battery, has proven to be useful in the neuropsychological assessment of cognitive deficits in patients with MCI-AD [21, 25]. Like the WMS, the CERAD battery combines multiple subtests which require individual interpretations. The CERAD Word List Memory subtest of the larger CERAD battery is presented to the participants in three trials [25]. At every 2 seconds, participants are required to read each word aloud. At the end of the reading task, participants would recall as many words as possible from the list in a single trial. Each trial has a maximum of 10 correct answers summing up to 30 correct answers in the three trials. Different scores can be calculated from the trials, including delayed recall and recognition, among other varying predefined scores [25]. In the same manner, the BNT is commonly used to access language-related cognitive deficits in MCI-AD and other neuropsychological disorders using confrontational word retrieval technique [26]. Both the 30-item BNT and 60-item BNT are commonly used to assess naming difficulties among patients with cognitive deficits. Patients are asked to say the names of specific images with a period of 20 seconds between multiple trials.

While the above test battery has been proven clinically useful [16, 20, 27, 28], we hypothesized that an independent linguistic battery comprising of only the language components or subtests of these popular test batteries could give better clinical diagnosis for the MCI-AD compared to using an exhaustive battery of tests. As such, we combined multiple clinical datasets and performed Exploratory Factor Analysis (EFA) to extract relevant language-based subtests from a combination of the CERAD word list, WLM language subtests, and the BNT subtests. Furthermore, while the EFA identified variables that show the underlying structure of the data, we trained a machine-learning algorithm that validates the clinical relevance of the independent linguistic battery for diagnosing MCI-AD using the loaded variables from the EFA.

## Methods

### Datasets

We used two datasets in this study. We obtained the first dataset from the Layton Aging and Alzheimer’s Disease Center and the Oregon Center for Aging and Technology Research Repository (http://www.ohsu.edu/xd/research/centers-institutes/orcatech/index.cfm), which is part of an existing study on MCI and AD at the Oregon Health and Science University (OHSU). The second dataset is based on the National Alzheimer’s Coordinating Center(NACC) Uniform Data sets version 3.0 (UDS 3.0)(https://www.alz.washington.edu/WEB/data_descript.html).

The OHSU study used a battery of tests like the CERAD battery (i.e., CDR, MMSE, CERAD Word List, WLM I & II, WAIS-R, and the BNT) to follow participants over a longitudinal period with at least a 6-month interval. The dataset consists of 34 healthy control individuals without any cognitive impairments and a matching 34 individuals with MCI. There were no significant age differences between the control and the MCI participants in that study. The individuals with MCI were diagnosed based on individual scores from the used battery of tests, including the CDR, Collateral CDR, and MMSE scores. A CDR score of 0 corresponds to the absence of MCI, while a CDR score of 0.5 or more and an MMSE score below 24 indicate the likelihood of MCI.

We extracted participants’ scores from the WLM subtests, CERAD Word List subtest, and the BNT subtests from the dataset. We extracted ten language-based items scores (*wordlist used, wordlist cannot read, wordlist trial 1, wordlist trial 2, wordlist trial 3, wordlist acquisition, wordlist intrusions, wordlist delayed recall, wordlist delayed intrusions, wordlist list recognition*) from the CERAD word list. Also, we extracted both the WLM I and WLM II scores from the dataset. More importantly, as at the time of conducting the OHSU study, the WMS-III battery was the existing version on which the WLM I and II were based.

The NACC UDS is based on data from 34 Alzheimer’s disease Centers founded by the National Institutes of Health. The dataset consists of multiple subject visits over ten years beginning from 2005. For this study, we extracted data from the third visit only since it contains unique participants with a sufficiently large number balanced across the MCI-AD diagnosed patients and the matching healthy control individuals. There were 197 MCI-AD and 270 cognitive healthy unique subjects in the third visit. Upon removing observations with non-relevant responses such as *unknown*, there were 178 MCI-AD and 270 cognitive healthy unique subjects remaining in the dataset.

For this study, we combined both the OHSU dataset and the NACC UDS dataset into a single dataset. Both datasets share the same set of variables as required for the linguistic battery apart from the ten CERAD wordlist items which are only present in the OHSU dataset. As such, we used multiple imputation techniques to construct a complete dataset with all the relevant variables [29]. Multiple imputation techniques have become popular in clinical research [29, 30]. The technique allows for filling in missing data from the observed data. More importantly, multiple imputations introduced uncertainties about the missing data through multiple iterations (bootstrapping) of generating different datasets based on the predictive distribution of the observed data [31]. In the end, the generated datasets are harmonically combined to obtain a single and plausible dataset [30]. We performed ten iterations of multiple imputations with a random seed of 54321 to impute the ten CERAD word list variables for the NACC UDS data based on the OHSU data. The multiple imputation process generated a total of 4,480 observations, which is the number of the original observations in ten places. Note that because the imputed data were either missing at random (MAR) or missing completely at random (MCAR) as a result of the combination of multiple datasets [30], there is substantial evidence in the literature that the multiple imputation techniques effectively reduce bias even with a large proportion of missing data [32, 33].

### Analysis

We divided the OHSU dataset into MCI-AD and Control groups. We performed Exploratory Factor Analysis (EFA) to extract latent linguistic constructs from the combined imputed dataset [34]. The EFA was performed on each independent group (i.e., MCI-AD and healthy control) to show the underlying constructs in each group and determine whether the constructs could adequately characterize the presence or absence of MCI-AD. Furthermore, the EFA shows the validity of the underlying constructs to the diagnosis of MCI-AD or otherwise. We based the validity evidence on the internal structure matrix of the EFA and a reliability measure of the internal consistency between the underlying constructs and the linguistic variables. The Principal Axis Factoring (PAF) was used as the extraction method for the EFA since all the variables do not have a normal distribution [35]. We specified the varimax orthogonal rotation to produce an uncorrelated factor in order to identify all possible underlying linguistic constructs [35]. For both the MCI-AD and healthy control groups, three factors were suggested to be appropriate by a scree test. A 0.4 cut-off point was set to identify variables that sufficiently load on each factor [29]. We excluded variables that loaded on multiple factors in the interpretation of the results.

A bivariate correlation analysis was performed to show correlations between the underlying linguistic constructs and all the variables using the Spearman correlation coefficient [36]. The purpose was to show the degree of relationship between the linguistic variables and the underlying constructs of MCI-AD and control groups. Furthermore, to ascertain internal consistency between the variables, we measured the reliability of the linguistic battery for differentiating between patients with MCI-AD and healthy controls using Cronbach’s alpha (*α*) coefficient [37].

Finally, for clinically diagnosed patients with MCI-AD, variables which loaded on the linguistic constructs from the EFA were used to train Support Vector Machines (SVM) algorithm [38], which is one of the famous and most robust machine learning algorithms [39]. We measured the performance of the machine learning algorithm using the Area Under the receiver operating characteristics (ROC) Curve (AUC) [40, 41]. The AUC is famous for evaluating the performance of clinical diagnostic and predictive models [42]. The AUC makes a tradeoff between the sensitivity (true positive rate) and the specificity (true negative rate) [40]. The percentage of positive and accurately classified observations is known as sensitivity. On the other hand, the specificity computes the percentage of negative observations which were accurately classified as negative. When the sensitivity of a classifier is 0.0, and the specificity is 1.0, then the confidence score of the diagnostic test is below the set threshold [11]. Conversely, when the specificity is 0.0, and sensitivity is 1.0, it means the confidence score of the diagnostic test is above the set threshold. A random diagnostic test has an AUC of 0.5 with a diagonal line connecting the origin (0, 0) to the final point (1, 1). An AUC of 1.0 is a perfect diagnostic test that ranks all positive observations above all negative observations [40]. While different clinical diagnostic scenarios make different tradeoff with the AUC, the recommended AUC for clinical purposes is 75 and above [15, 43].

Statistical analyses (EFA and correlations) were performed using the Statistical Analysis Software (SAS) version 9.4. The machine learning experiments and evaluation were performed in RStudio version 1.1.463 using the e1071 package for the SVM experiments [38], pROC package for the AUC evaluations [42], and the gplots package for the heatmaps [45].

## Results & discussion

### Summary statistics

Table 1 shows the summary statistics of the combined dataset before the multiple imputations were performed. The summary excludes the CERAD word list variables as they were not part of the NACC UDS data. The number of observations in each group excludes observations with at least one missing value. Compared to the male patients, there was a higher number of female patients in both the MCI-AD (58.8%) and the control groups (58.2%). About 91% of the MCI-AD were whites, while around 93% of the healthy controls were whites. Surprisingly, the MCI-AD group had, on average, more years of education (16.21±9.05) compared to the healthy control group (15.50±6.07), however, the difference is not statistically significant. Also, there was no statistically significant difference between the mean age of the MCI-AD (85.39±7.56) and the control (84.18±6.91) group. CDR, LMI, and LMII had a statistically significant difference between the MCI-AD and control groups. On average, the CDR was higher in the MCI-AD (0.12±0.22) compared to the control group (0.05±0.17). Compared to the MCI-AD group, the control group had higher LMI and LMII values on average. There was no statistically significant difference between the MCI-AD and control groups for the MMSE and Boston variables. Note that the BNT variable is referred to as Boston in both datasets.

**Table 1:** Summary statistics for the combined dataset before multiple imputations. The number of observations is shown for each category of sex and race. Mean (standard deviation) is shown for all other variables. *n* excludes observations with missing values.

Variable	MCI-AD (n = 187)	Control (n = 261)	*p*-value
SEX (Male/Female)	77/110	109/152	0.9015
RACE(White/Black/Asian)	170/16/0	243/17/1	0.3143
EDUCATION (Years)	16.21(9.05)	15.50(6.07)	0.3514
AGE (Years)	85.39(7.56)	84.18(6.91)	0.0795
CDR	0.12(0.22)	0.05(0.17)	<0.0000
MMSE	27.18(2.05)	27.56(2.85)	0.1000
LM I	10.64(4.39)	11.51(4.76)	0.0500
LM II	8.88(4.82)	9.89(5.27)	0.0400
BOSTON	25.35(3.43)	25.85(3.74)	0.0900

Table 2 shows the summary statistics of the imputed datasets for the MCI-AD and control groups from the ten iterations of multiple imputations. Among the demographic variables, there was no statistical significance between male and female patients. Most of the CERAD word list variables were statistically significant, except *wordlistcantread*, *wordlistintrusions*, and *wordlistrecognition*.

**Table 2:** Summary statistics of the imputed datasets for the MCI-AD and control groups from the ten iterations of multiple imputations. Mean (standard deviation) is shown for all other variables.

Variable	MCI-AD (n = 1870)	Control (n = 2610)	*p*-value
SEX	1.59 (0.49)	1.58(0.49)	0.6948
RACE	1.61(7.15)	1.08(0.35)	0.0014
EDUCATION (Years)	16.21(9.03)	15.50(6.06)	0.0031
AGE (Years)	85.39(7.54)	84.18(6.90)	<0.0001
CDR	0.12(0.22)	0.05(0.17)	<0.0001
MMSE	27.18(2.05)	27.56(2.85)	<0.0001
LM I	10.68(4.38)	11.51(4.75)	<0.0001
LM II	8.93(4.81)	9.89(5.26)	<0.0001
BOSTON	25.38(3.41)	25.94(3.73)	<0.0001
WORDLISTUSED	1.43(0.74)	1.48 (0.59)	0.0169
WORDLISTCANTREAD	0.12(2.00)	0.05(1.58)	0.2012
WORDLISTTRIALI	4.51(2.73)	4.83(1.99)	<0.0001
WORDLISTTRIALII	6.16(2.40)	6.51(1.89)	<0.0001
WORDLISTTRIALIII	6.93(2.68)	7.27(1.88)	<0.0001
WORDLISTACQUISITION	17.67(6.12)	18.67(4.81)	<0.0001
WORDLISTINTRUSIONS	0.61(2.85)	0.64(1.42)	0.6174
WORDLISTDELAYEDRECALL	5.37(3.35)	5.89(2.53)	<0.0001
WORDLISTDELAYEDINTRUSIONS	0.36(1.04)	0.21(0.80)	<0.0001
WORDLISTRECOGNITION	19.16(4.80)	19.08(1.64)	0.4592

### Underlying linguistic constructs with exploratory factor analysis

Table 3 shows the underlying linguistic constructs for patients with MCI-AD. On that group, the logical memory subtests and the CERAD wordlist subtests loaded on the three factors. We observed that the MCI-AD group could be characterized as having linguistic deficits that can be measured by different linguistic constructs. We identified three different linguistic themes based on the variables that uniquely loaded on each of the factors. Most of the loaded variables have communalities above 70%, which shows a substantial amount of each variable’s variance that is explainable by the factors [46].

**Table 3:** EFA structure matrix for the MCI-AD group. Uniquely loaded variables (>0.40) are marked with asterisks. Important *r* and communality (Comm.) values are boldfaced.

Variable	*r*	Factor1	Factor2	Factor3	Comm.
SEX	0.17	14	6	7	0.03
RACE	0.10	85*	-33	-22	**0.88**
EDUCATION (Years)	-0.27	-39	-23	-19	0.24
AGE (Years)	0.24	-15	-16	25	0.11
CDR	-0.41	-9	-25	-5	0.08
MMSE	0.30	0	21	36	0.17
LM I	**0.92**	4	8	89*	**0.81**
LM II	**0.93**	5	7	91*	**0.83**
BOSTON	0.30	0	23	22	0.10
WORDLISTUSED	0.26	-2	2	22	0.05
WORDLISTCANTREAD	-0.27	-39	26	-2	0.22
WORDLISTTRIALI	0.71	73	51	6	0.80
WORDLISTTRIALII	**0.82**	-23	87*	12	**0.82**
WORDLISTTRIALIII	**0.79**	0	87*	1	**0.76**
WORDLISTACQUISITION	**0.95**	25	95*	8	**0.96**
WORDLISTINTRUSIONS	**-0.52**	-85*	-6	6	**0.72**
WORDLISTDELAYEDRECALL	**0.74**	74*	35	11	**0.68**
WORDLISTDELAYEDINTRUSIONS	-0.33	-15	-3	-43*	**0.20**
WORDLISTRECOGNITION	**0.55**	92*	-11	-10	**0.87**

Factor 1 represents a *linguistic translation* construct (wordlist recognition, wordlist recall, and wordlist intrusion) that shows the impaired ability of the patients to perform recognition and recall processes with a certain level of intrusion or disturbances during that process. Since the combined dataset consists of a predominantly white population, we can only infer that the linguistic translation construct might be specific to patients with MCI-AD who are whites. Factor 2 shows the evidence of *linguistic retention* construct (wordlist learning trials II, III, wordlist acquisition), which shows the inability of patients with MCI-AD to learn and retain certain linguistic components successfully. Finally, Factor 3 implies the evidence of *linguistic transient* construct as observed in the immediate and delayed components of the logical memory (LMI and LMII) subtests with the negatively loaded wordlist delayed intrusions.

We believe these three linguistic themes (*linguistic translation*, emphlinguistic retention, and *linguistic transient*) speak to the non-trivial nature of diagnosing the MCI-AD group. At the same time, we believe our analysis uncovers the fact that no single underlying construct can characterize the complicated nature of MCI-AD [2, 13]. As such, these multiple linguistic constructs could be used in a linguistic battery that captures essential linguistic biomarkers for identifying patterns of impaired speech that is specific to patients with MCI-AD [6, 8, 11, 12, 15].

Table 4 shows the underlying linguistic construct for the healthy control group. Unlike the MCI-AD group, the control group loaded differently, albeit with three different underlying constructs as observed in the MCI-AD group. Also, like the MCI-AD group, most of the loaded variables showed communalities above 70%, which shows a substantial amount of each variable’s variance that is explainable by the factors [46]. Factor 1 showed combined evidence of *linguistic translation* and *linguistic retention* constructs. This forms the *translate-retention* construct (wordlist delayed recall, wordlist learning trials II, III, wordlist acquisition). The *translate-retention* construct characterizes the difficulty of differentiating patients with MCI-AD from healthy control individuals since many cognitive healthy individuals have been shown to share overlapping biomarkers with patients who have MCI-AD [8]. We believe the overlap between MCI-AD and healthy control emphasizes the non-trivial nature of diagnosing MCI-AD at the early stages. Factor 2 shows the evidence of *linguistic competence* (MMSE, LMI, LMII, Boston, and *wordlistused*) construct as most healthy controls tend to do very well with the MMSE, logical memory subtests, the Boston naming test, and the number of words used. The CDR, on the other hand, is famously sensitive to AD of Dementia-type and even less sensitive to MCI-AD, which could be a reason why it did not load on the MCI-AD. Unlike the control group, the MCI-AD group did not load on the MMSE, Boston, and *wordlistused* variables. Finally, Factor 3 shows a certain level of *linguistic intrusion* construct (wordlist intrusions and wordlist delayed intrusions). It is expected that some of the healthy controls would load on the linguistic intrusion since both MCI-AD and the control groups are likely to have similar responses to linguistic disturbances [2, 13].

**Table 4:** EFA structure matrix for the control group. Uniquely loaded variables (>0.40) are marked with asterisks. Important *r* and communality (Comm.) values are boldfaced.

Variable	*r*	Factor1	Factor2	Factor3	Comm.
SEX	-0.18	3	-9	-16	0.04
RACE	-0.26	-12	-22	-12	0.08
EDUCATION (Years)	0.43	-1	45	75	0.76
AGE (Years)	-0.08	-25	2	-9	0.07
CDR	-0.36	-37	-39	11	0.30
MMSE	**0.56**	32	66*	-22	**0.59**
LM I	**0.83**	13	82*	-7	**0.69**
LM II	**0.88**	11	84*	-9	**0.72**
BOSTON	**0.51**	37	54*	-7	**0.44**
WORDLISTUSED	**0.53**	3	59*	0	**0.34**
WORDLISTCANTREAD	-0.11	2	-12	-3	0.01
WORDLISTTRIALI	0.73	72	16	-42	0.73
WORDLISTTRIALII	**0.83**	84*	23	4	**0.77**
WORDLISTTRIALIII	**0.79**	81*	-1	-17	**0.69**
WORDLISTACQUISITION	**0.94**	94*	15	-23	**0.96**
WORDLISTINTRUSIONS	0.50	-19	-23	51*	**0.35**
WORDLISTDELAYEDRECALL	**0.63**	67*	29	-36	**0.66**
WORDLISTDELAYEDINTRUSIONS	**0.76**	6	-13	82*	**0.70**
WORDLISTRECOGNITION	-0.59	43	21	-64	0.64

### Correlation between variables and the underlying linguistic constructs

As shown in Tables 3 and 4, all the loaded variables showed moderate to very strong Spearman correlation coefficients with the identified factors. This shows that the variables are effective in characterizing either the MCI-AD group or the healthy control group. More importantly, we observed many variables with very strong positive correlations in the MCI-AD group compared to the control group. For example, the LMI and LMII variables in the MCI-AD group have Spearman correlation coefficients of 0.92 and 0.93, respectively, compared to 0.83 and 0.88 in the healthy control group. We believe these results indicate the difference in the linguistic deficits between the MCI-AD group and the control group. Other loaded variables showed similar correlation coefficients in both the MCI-AD and control groups.

Also, the between factor correlations showed the difference between the underlying linguistic constructs since we specified the varimax orthogonal rotation to produce an uncorrelated factor. On the MCI-AD group, Factor 1 had a 51% (p<0.0001) positive correlation with Factor 2 and a 38% (p<0.0001) positive correlation with Factor 3. Factor 2 had a non-significant and marginal -0.004 (p = 0.8501) correlation with Factor 3. On the healthy control group, however, Factor 1 had a non-significant 2% (p = 0.3420) negative correlation with Factor 2 and a 4% (p = 0.0405) positive correlation with Factor 3. Finally, Factor 2 had a 21% (p<0.0001) negative correlation with Factor 3. Again, we believe the very weak to moderate correlations which were observed between the extracted factors in the MCI-AD group shows the difficulty in effectively diagnosing MCI-AD because of its complicated pattern of biomarkers [2, 13]. The control group, however, demonstrated a clear pattern of uncorrelated underlying linguistic patterns.

A standardized Cronbach’s alpha of 0.74 was achieved with ten variables from the MCI-AD group (LMI, LMII, Boston *wordlistused*, *wordlisttrialI*, *wordlisttrialII*, *wordlisttrialIII*, *wordlistacquisition*, *wordlistdelayedrecall*, and *wordlistrecorgnition*). On the control group, we realized a standardized Cronbach’s alpha of 0.87 for the same set of variables. We believe that a Cronbach’s alpha of 0.74 on the MCI-AD group showed moderate reliability for this exploratory phase of the study. At the same time, a Cronbach’s alpha of 0.87 on the control group emphasizes the observed difference between the MCI-AD and the healthy control groups. The ten variables identified by Cronbach’s alpha form our independent linguistic battery to be evaluated with machine learning techniques.

Finally, Table 5 shows the Fraction of Missing Information (FMI) and Relative Efficiency (RE) for the linguistic variables. Although the proportion of missing data for the variables used in the MI process is approximately 87%, the FMI and RE are better measures that demonstrate the benefits and efficiency of the MI process [32]. More importantly, each linguistic variable shows a relative efficiency above 90%, which indicates an effective reduction in bias even when the proportion of missing data is large.

**Table 5:** Fraction of Missing Information (FMI) and Relative Efficiency (RE) for linguistic variables.

Variable	FMI	RE
LM I^a^	0.0156	0.9984
LM II^a^	0.0094	0.9991
Boston^a^	0.0229	0.9977
WORDLISTUSED	0.8723	0.9198
WORDLISTTRIALI	0.7855	0.9272
WORDLISTTRIALII	0.8466	0.9220
WORDLISTTRIALIII	0.8550	0.9212
WORDLISTACQUISITION	0.7969	0.9262
WORDLISTDELAYEDRECALL	0.7293	0.9320
WORDLISTRECOGNITION	0.6322	0.9405

^a^ Component of Linguistic Battery II in Tables 7 & 8.

### Evaluation of the linguistic battery with machine learning techniques

To automate the diagnosis of the MCI-AD from healthy control patients [24], we performed different sets of experiments to verify the hypothesis that an independent linguistic battery could better diagnose patients with MCI-AD compared to the MMSE, CDR, or a combination of the MMSE and CDR test battery put together. As such, we build machine learning models using only the ten variables that loaded in the EFA process and further confirmed reliable by Cronbach’s alpha.

We verified the importance of covariates in diagnosing patients with MCI-AD. We experimented with and without the four covariates (age, sex, race, and education). We also evaluated the Linguistic Battery model with and without the CERAD word list.

Each model in our experiment was tuned to the best SVM parameters on a separate 1840 random observations from the total 4840 imputed observations. Consistent with the literature, our tuning process used 10-fold cross-validation that ensured optimal parameters for each model [47]. We used the SVM and tune functions in the e1071 R library to perform the tuning process [38]. The SVM kernel was set to the Radial kernel, the cost parameters range from 10^-1^ to 10^2^, and the gamma parameter was set to be selected from a default list of 0.5, 1, and 2 parameter values. Using the optimal parameters, the remaining 3000 observations were used in the final classification for generating the AUC with 10-fold cross-validation. Also, it is worth mentioning that other variants of the SVM algorithm such as the Recursive Feature Elimination (RFE) [48], could be used to identify useful features or build classification models without the EFA technique. However, our goal was to employ an explainable method of analyses parallel to the more complex SVM algorithm. Table 6 shows the identified optimal SVM parameters for each model.

**Table 6:** Identified optimal SVM parameters for each model.

Model	Kernel	Cost	Gamma
Linguistic Battery I	Radial	10	2
Linguistic Battery II	Radial	1	1
MMSE	Radial	0.1	0.5
CDR	Radial	100	1
MMSE & CDR	Radial	100	1
All combined	Radial	1	2
Linguistic Battery I w/ covariates	Radial	10	1
Linguistic Battery II w/ covariates	Radial	1	0.5
MMSE w/ covariates	Radial	1	1
CDR w/ covariates	Radial	0.1	2
MMSE & CDR w/ covariates	Radial	1	2
All combined w/ covariates	Radial	10	2

* Linguistic Battery I is with CERAD wordlist. Linguistic Battery II excludes CERAD wordlist.

In the first experiment, we trained an SVM model using all the linguistic variables with the best Cronbach’s alpha from our correlation analysis to form the independent linguistic battery I (LMI, LMII, Boston, *wordlistused*, *wordlisttrialI*, *wordlisttrialII*, *wordlisttrialIII*, *wordlistacquisition*, *wordlistdelayedrecall*, and *wordlistrecorgnition*). Second, we trained an SVM model with all the linguistic variables except the CERAD word list variables to form an independent linguistic battery II (LMI, LMII, Boston). Third, we trained an SVM model with the MMSE variable. Fourth, we trained an SVM model with the CDR variable. Fifth, we trained an SVM model with a combination of the MMSE and the CDR variables. Finally, we trained an SVM model with a combination of the best of linguistic battery I and II, MMSE, and CDR variables.

Table 7 shows the results of the models without the four covariates. More often than not, the MMSE and the CDR are interpreted independent of the covariates used in this study (i.e., age, sex, race, education). Our results show that these covariates could contribute to the effectiveness of the diagnosis of MCI-AD, and thus, should be considered in the context of interpreting the results. Nevertheless, without the covariates, the linguistic battery I showed better AUC of 0.72 (CI: 0.70-0.73, p<0.0001) and linguistic battery II showed better AUC of 0.88 (CI: 0.86-0.89, p<0.0001) compared to an AUC of 0.59 for the MMSE; 0.55 for CDR; and 0.64 for the combination of MMSE and CDR. These results support the findings in [14, 16, 20], which found limited evidence that the MMSE could be used to clinically diagnose MCI-AD. Using the best linguistic battery, we recorded a 29% improvement on the MMSE, 33% improvement on the CDR, and a 24% improvement on the combination of MMSE and CDR. Unlike the MMSE and the CDR, we see that the linguistic battery with or without the CERAD word list is robust to achieve a clinically reliable AUC for diagnosis even when the covariates are not being considered at all. We believe that the combination of the linguistic battery with MMSE and CDR improves the AUC of the combined test battery put together by 34%. Overall, when compared to MMSE and CDR, our results show the linguistic battery alone has the potential to effectively diagnose patients with MCI-AD without controlling for age, sex, race, and education.

**Table 7:** Machine learning diagnostic performance of models *without* covariates using the Area Under the ROC Curve (AUC)—(No covariates used in the models).

Model	AUC	CI	p-value
Linguistic Battery I	0.72	0.70-0.73	<0.0001
Linguistic Battery II	0.88	0.86-0.89	<0.0001
MMSE	0.59	0.57-0.62	<0.0001
CDR	0.55	0.51-0.58	<0.0001
MMSE & CDR	0.64	0.62-0.66	<0.0001
All combined	0.98	0.97-0.98	<0.0001

* Linguistic Battery I is with CERAD wordlist. Linguistic Battery II excludes CERAD wordlist.

Table 8 emphasizes the importance of covariates in diagnosing patients with MCI-AD. The table shows the AUC comparison between the models with covariates. When controlled for age, sex, race, and education, the linguistic batteries I and II gave robust AUC values of 0.84 (CI: 0.83-0.86, p<0.0001) and 0.97 (CI: 0.96-0.97, p<0.0001), which demonstrates the effectiveness of the linguistic battery in identifying linguistic biomarkers in patients with MCI-AD. Compared to using the MMSE alone, the best linguistic battery had a better diagnostic performance by 20%. Similarly, compared to using the CDR alone, the best linguistic battery showed better diagnostic performance by 28%. Even when both MMSE and CDR are combined, the best linguistic battery had better performance by 10%. The combination of the linguistic battery with MMSE and CDR showed showed that the linguistic battery improves the performance of a combination of MMSE and CDR by 13%. Also, when the covariates are included, the CDR and MMSE actually do perform much better diagnostically. At the same time, even though the combination of all measures gives an almost perfect AUC, using the linguistic battery alone can lead to effective and efficient screening process that avoids the rigor of having to combine the MMSE and the CDR especially for screening through a large population [2, 20].

**Table 8:** Machine learning diagnostic performance of models *with* covariates using the Area Under the ROC Curve (AUC)—(Models include covariates).

Model	AUC	CI	p-value
Linguistic Battery I w/ covariates	0.84	0.83-0.86	<0.0001
Linguistic Battery II w/ covariates	0.97	0.96-0.97	<0.0001
MMSE w/ covariates	0.77	0.75-0.78	<0.0001
CDR w/ covariates	0.68	0.66-0.71	<0.0001
MMSE & CDR w/ covariates	0.86	0.85-0.88	<0.0001
All combined w/ covariates	0.99	0.99-1.00	<0.0001

* Linguistic Battery I is with CERAD wordlist. Linguistic Battery II excludes CERAD wordlist.

Although the Wechsler LMI, LMII, and Boston variables appeared to be more effective in the linguistic battery compared to the imputed CERAD word list, benefits of the multiple imputation technique can be seen in the difference between linguistic battery I and the individual MMSE and CDR test batteries. In Table 7, the linguistic battery I showed a 13% improvement over MMSE, 17% improvement over CDR, and 8% over the combination of MMSE and CDR. Similarly in Table 8, the linguistic battery showed a 7% improvement over MMSE, 16% over the CDR, and only lost 2% to a combination of MMSE and CDR, which could easily be gained by using the linguistic battery II. Notably, we believe the multiple imputation technique has helped understand the underlying linguistic patterns that could help predict the presence of MCI-AD without using exhaustive test batteries.

Furthermore, we constructed heatmaps in the form of hierarchical clustering of the data. The heatmaps emphasize the sensitivity of the linguistic battery with covariates to capture the underlying difference between the MCI-AD and the healthy control groups.

**Fig 1:**
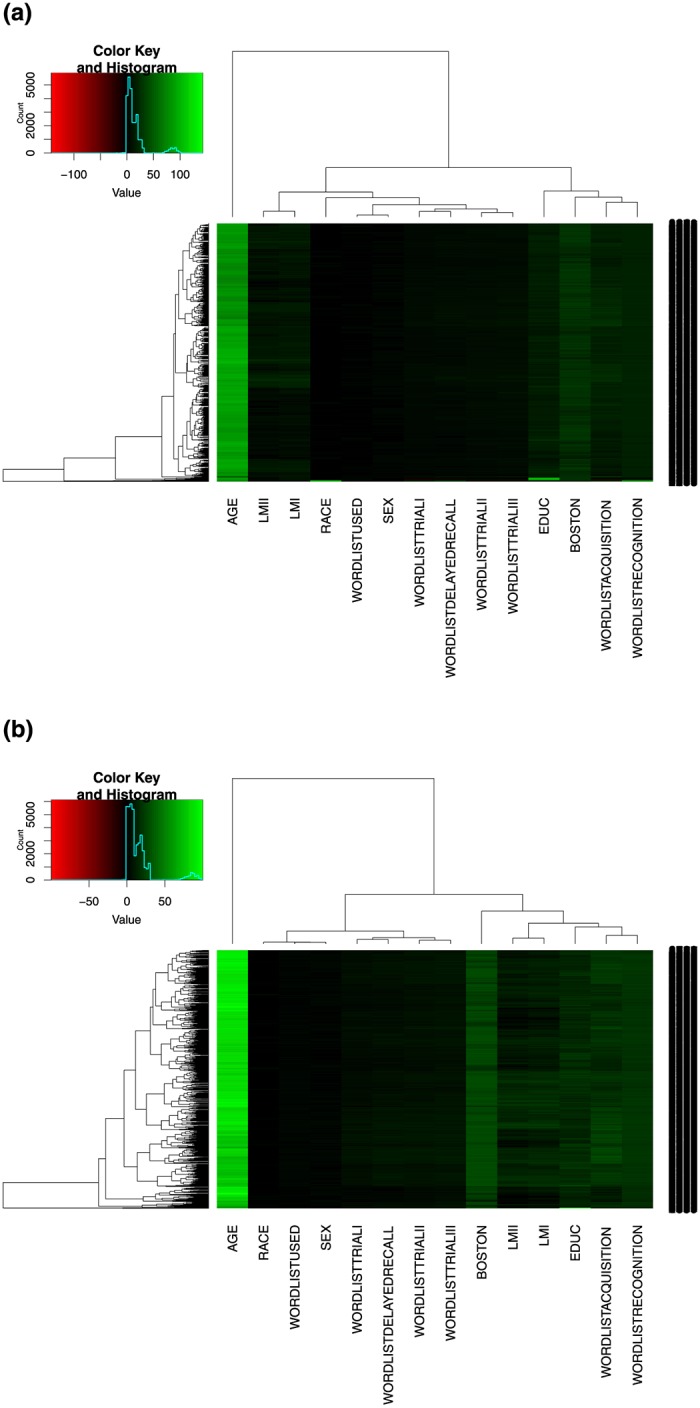
Comparison of the underlying patterns of the linguistic battery between the MCI-AD and healthy control groups. (**a**) A. MCI-AD group. (**b**) B. Healthy control group.

Fig 1a and 1b show the underlying patterns of the linguistic battery with covariates by the group. We observed a unique difference between the underlying patterns of the ten linguistic variables with Boston, LMI, LMII, *wordlistacquisition*, and *wordlistrecorgnition* variables showing distinct patterns in both heatmaps. Compared to the MCI-AD group, the healthy control group showed a lighter color gradient across the ten linguistic variables, an indication that the healthy control group performs very well with those variables. The unique contributions of the covariates were also emphasized in the heatmaps. Compared to the MCI-AD group, age and education variables showed a lighter color gradient in the healthy control group. This observation supports the result of the linguistic battery with covariates in Table 8, which shows improvement over the linguistic battery without covariates in Table 7.

**Fig 2:**
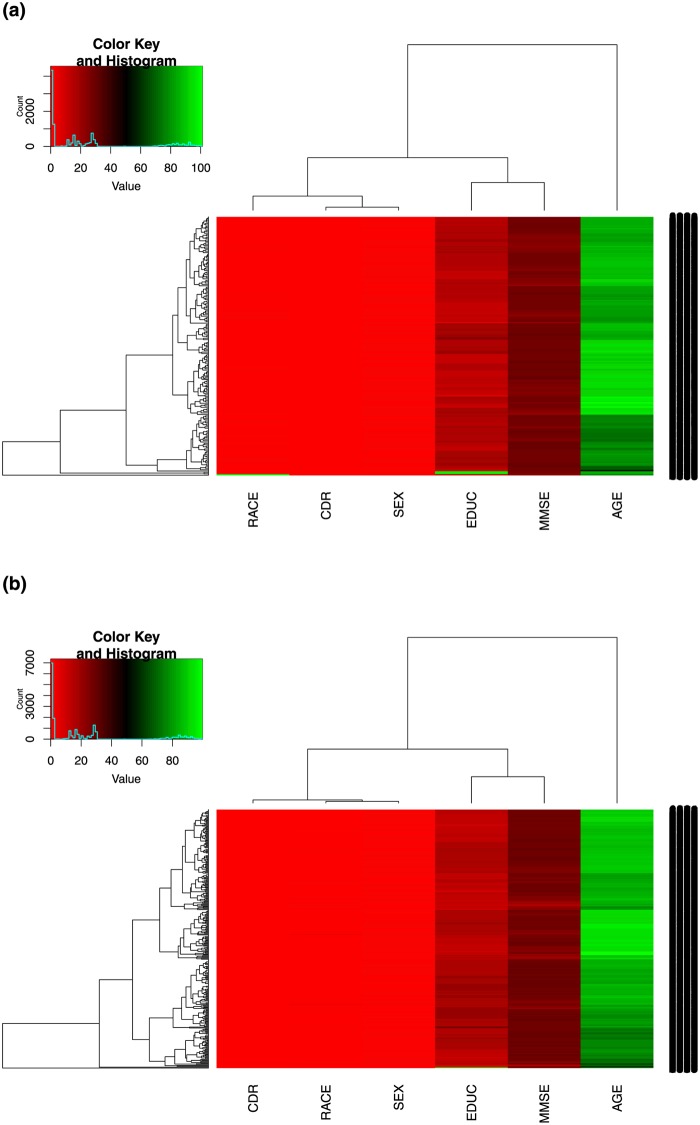
Comparison of the underlying patterns of combined MMSE and CDR test battery between the MCI-AD and healthy control groups. (**a**) A. MCI-AD group. (**b**) B. Healthy control group.

On the other hand, Fig 2a and 2b show the underlying patterns of the combined MMSE and CDR variables with the four covariates for MCI-AD and the healthy control group. Across the MMSE and CDR variables, we observed no distinct difference between the patterns of the MCI-AD and the healthy control group, an indication that the MMSE and CDR are less sensitive to differentiating patients with MCI-AD from cognitively healthy individuals. More importantly, we observed that the MMSE and CDR could not be effective without considering the covariates. A slight difference was observed across the age and education variables, which yet shows the importance of the covariates in administering neuropsychological tests, hence supporting the results of the models in Table 8.

Overall, a distinct difference was created by the linguistic battery variables between the MCI-AD and healthy control individuals. This is an indication that the linguistic battery can effectively show the difference between patients with MCI-AD and healthy control individuals, compared to the MMSE, CDR, or a combination of both.

## Limitations

One of the limitations of this study is the exploratory nature of the analysis, especially in identifying the underlying linguistic constructs. As a follow-up to the EFA, confirmatory factor analysis could be performed to validate the assumptions made in the EFA [34, 49].

Another limitation lies in the use of multiple imputation techniques for imputing the missing CERAD wordlist variables for the NACC UDS data. While multiple imputation techniques have been successful in clinical and epidemiological research [30, 31], there remain ongoing debates about its implication on the interpretation of findings [29]. We also recognize that using the third visit only from the NACC UDS dataset might vary the performance from the other study visits or the average over all the visits.

This study did not use objective measures such as neuroimages due to the absence of such measures in the datasets used in this study. Future works could include neuroimages in addition to the linguistic battery for clinically diagnosing MCI-AD.

Also, the majority-white dataset is another limitation in this study. There is the possibility that the results may be associated with the demographics of that population alone. Future works could consider a dataset with even distribution of the race/ethnicity and other demographic variables to measure their actual impact on the outcome.

Finally, the machine learning algorithm used for building the diagnostic models was tuned to the optimal parameters on each model [47]. As such, performing similar experiments on a different dataset would require that the machine learning algorithm is tuned on that dataset to avoid the pitfall of relying in part on the sample’s error variance structure generated by the machine learning algorithm in this study.

## Conclusion

Exploratory factor analysis and a machine learning evaluation of an independent linguistic battery for diagnosing Mild Cognitive Impairment due to Alzheimer’s disease have been investigated. The linguistic battery combines the language-based CERAD Word List subtests, Wechsler Logical Memory subtests, and the Boston naming test to distinguish the underlying linguistic construct of patients with MCI-AD from the healthy control individuals. The linguistic battery consists of ten linguistic variables with distinct underlying linguistic constructs achieving a Cronbach’s alpha of 0.74 on the MCI-AD group and 0.87 on the healthy control group. Also, we showed that the linguistic battery could be automated using a robust machine learning algorithm. The results of the machine learning evaluation using the clinically relevant AUC measure showed that the best linguistic battery gives a robust AUC of 0.97 when controlled for age, sex, race, and education. At the same time, our results show that the linguistic battery alone gives a robust diagnostic performance with a clinically reliable AUC of 0.88 without controlling for age, sex, race, and education. Overall, the linguistic battery showed a better diagnostic performance compared to MMSE, CDR, and a combination of MMSE and CDR.
